# A control strategy to investigate the relationship between specific productivity and high-mannose glycoforms in CHO cells

**DOI:** 10.1007/s00253-016-7380-4

**Published:** 2016-02-24

**Authors:** Dénes Zalai, Helga Hevér, Krisztina Lovász, Dóra Molnár, Patrick Wechselberger, Alexandra Hofer, László Párta, Ákos Putics, Christoph Herwig

**Affiliations:** Department of Biotechnology, Gedeon Richter Plc., 19-21, Gyömrői út, Budapest, 1103 Hungary; Institute of Chemical Engineering, Research Area Biochemical Engineering, Vienna University of Technology, Gumpendorfer Strasse 1a, 1060 Vienna, Austria; Spectroscopic Research Department, Gedeon Richter Plc., 19-21, Gyömrői út, Budapest, 1103 Hungary; CD Laboratory for Mechanistic and Physiological Methods for Improved Bioprocesses, Vienna, Austria

**Keywords:** Cell culture, CHO, Product quality attributes, Monoclonal antibody, Control of specific productivity, Glycosylation

## Abstract

The integration of physiological knowledge into process control strategies is a cornerstone for the improvement of biopharmaceutical cell culture technologies. The present contribution investigates the applicability of specific productivity as a physiological control parameter in a cell culture process producing a monoclonal antibody (mAb) in CHO cells. In order to characterize cell physiology, the on-line oxygen uptake rate (OUR) was monitored and the time-resolved specific productivity was calculated as physiological parameters. This characterization enabled to identify the tight link between the deprivation of tyrosine and the decrease in cell respiration and in specific productivity. Subsequently, this link was used to control specific productivity by applying different feeding profiles. The maintenance of specific productivity at various levels enabled to identify a correlation between the rate of product formation and the relative abundance of high-mannose glycoforms. An increase in high mannose content was assumed to be the result of high specific productivity. Furthermore, the high mannose content as a function of cultivation pH and specific productivity was investigated in a design of experiment approach. This study demonstrated how physiological parameters could be used to understand interactions between process parameters, physiological parameters, and product quality attributes.

**Electronic supplementary material:**

The online version of this article (doi:10.1007/s00253-016-7380-4) contains supplementary material, which is available to authorized users.

## Introduction

The amount of biopharmaceutical products expressed in mammalian cell lines has been constantly increasing in the last decade (Walsh 2014). Parallel to the success of mammalian expression systems, the scientific understanding of complex interactions between process parameters and product quality attributes in cell culture processes has been also expanding. This knowledge has become especially critical in biosimilar development, where critical quality attributes (CQAs) have to be steered in a tight range defined by the original product (McCamish and Woollett 2011). The swift scientific progress enabled to identify the production cells’ physiological attributes which ultimately determine the interactions between process input parameters and product quality (Carinhas et al. 2012; Dickson 2014). The successful integration of physiological knowledge into process control tools and their applicability to adjust product quality attributes have been recently reviewed (Zalai et al. 2015a).

A frequently investigated physiological parameter of recombinant cell culture processes is specific productivity (*q*_P_), which quantifies the rate of protein expression per cell and time unit. Product titer, a variable frequently defined as a key performance indicator, is affected by specific productivity to a great extent. Accordingly, the maximization of *q*_P_ is an important target of bioprocess development (Kou et al. 2011; Schaub et al. 2012; Templeton et al. 2013). Moreover, as specific productivity delivers time-resolved information on the kinetics of recombinant protein synthesis, it can be used to investigate the interactions between processing events, product formation, and changes in post-translational modifications (Sou et al. 2015). Accordingly, *q*_P_ is a key parameter to understand links between cell physiology and product quality. An important basis of this knowledge should be the mechanistic understanding of interactions between the rate of product formation and the progress of post-translational modifications.

To investigate mechanistic interactions between specific productivity and product quality, strategies to control *q*_P_ at multiple constant levels are required. As to our knowledge, approaches reported in the scientific literature exclusively targeted maximal *q*_P_ and did not aim to control this physiological parameter at different levels. The reported approaches either used genetic engineering to enhance protein expression (Kober et al. 2013; Seth et al. 2007; Xiao et al. 2014) or applied process control strategies such as cell cycle arrest (Du et al. 2015) or medium development (Kang et al. 2012; Sellick et al. 2011) to increase product titer. The latter strategy is based on the recognition that a limitation of key nutrients (e.g., amino acids) leads to a decrease in specific productivity, which can be restored by the supplementation of these substances (Lu et al. 2013; Read et al. 2013; Sellick et al. 2011). However, these results also suggest that a targeted limitation and the subsequent continuous feeding of the limiting amino acids can be used to control specific productivity in fed-batch processes. The addition of nutrients in limiting amounts has been already applied in cell culture processes to adjust another physiological parameter, specific growth rate. Aehle et al. added glutamine by using a simple open-loop control and successfully controlled the specific growth rate of the cells at four different levels (Aehle et al. 2011a). The same authors also developed a closed-loop control based on oxygen uptake rate (OUR) to control specific growth rate at a constant level for a long time period (Aehle et al. 2012). These studies already demonstrated the controllability of physiological parameters in cell culture processes. However, the control of specific productivity at multiple levels has not been reported previously in the literature to our knowledge. The importance of *q*_P_ control strategies has been already postulated by Hossler et al., stating that hypothesized links between the rate of product formation and product glycosylation can be only verified by controlling specific productivity at multiple levels (Hossler et al. 2007).

In this case study, we demonstrate the use of specific productivity as a control parameter in a fed-batch CHO process. The OUR was used to monitor the metabolic activity of the culture and to detect the onset of nutrient limitations. Based on this on-line signal, a feeding strategy was developed to obtain different *q*_P_ profiles. The control of *q*_P_ at different levels allowed investigating interactions between the rate of product formation and post-translational modifications such as product glycosylation.

## Materials and methods

### Cell line and pre-culture

Suspension cultures of two CHO-K1-derived cell lines (referred to as cell lines A and B) expressing the same IgG1 monoclonal antibody were maintained in shake flasks before inoculating the bioreactors. Stocks were revived in commercially available basal medium (ActiCHO P, GE Healthcare, UK), supplemented with 8 mM L-Gln and 5 mg/l insulin. The cells were sub-cultured every 3–4 days with a seeding density of 0.3 · 10^6^ cells/ml and were grown in shake flasks of different scales. The shake flasks were incubated at 37 °C with humidified air containing 5 % CO_2_ and agitated at 100-rpm orbital shaking.

### Bioreactor cultivations

Fed-batch cultivations were performed in bioreactors with 1 l maximal working volume (Sartorius AG, Germany). The targeted seeding cell density was 0.5 · 10^6^ cells/ml. Temperature, pH, and pO_2_ were controlled by a Biostat BPlus Twin DCU (Sartorius AG, Germany). Stirring speed was set to 125 rpm, initial cultivation temperature was 37 °C, initial pH setpoint was 7.2, and the dissolved oxygen rate was maintained at 40 % of air saturation by air–oxygen mixture sparging. The pH value was controlled at the current setpoint ±0.02 by automatic addition of 10 % H_3_PO_4_ solution or 0.5 M Na_2_CO_3_ solution. The shifting of pH and temperature was performed on cultivation days 3 and 5, respectively. Temperature was shifted to 33 °C, and pH was shifted to pH 6.9. In the design of experiment (DoE) experiments, pH was shifted to the pre-defined setpoint according to the experimental design (see Fig. 7a). The basal medium was the same as the one used for the shake flask pre-culture cultivations (vide supra).

#### Feeding strategy

The feed medium was a proprietary medium. Bolus feeding was initiated on the third cultivation day by adding a pre-defined amount of feed medium to the culture broth at a high feeding rate. Continuous feeding was carried out by applying Watson Marlow 120U and 101U/R pumps (Watson Marlow, UK) and PharmMed BPT pump tubings (Saint-Gobain Performance Plastics, France). The supplementary feed was a proprietary feed medium containing high concentrations of essential amino acids dissolved at alkaline pH. Each pump and tubing combination was tested at several pump speed setpoints to obtain pump speed–feeding rate calibrations. These calibrations were subsequently used during the experiments to adjust feeding rates to the pre-defined setpoints. Feeding rates were also determined gravimetrically during the experiments, and pump speed was adjusted when required.

### Measurements

#### Real-time measurements

The bioreactors and feed mediums were placed on balances (Mettler Toledo, Switzerland) to determine broth and liquid volumes gravimetrically. The balance signals as well as on-line measured process parameters (pH, pO_2_, and temperature) were collected by the Biostat BPlus DCUs and processed in a CitectSCADA system (Schneider Electric, Rueil-Malmaison, France) via local area network connection. Capacitance of the cultures was measured with Biomass Monitor 220 (Aber Instruments, Aberystwyth, UK) using 12-mm annular sensors. OUR was determined with the stationary liquid phase method as described in the literature (Ruffieux et al. 1998). The temperature dependence of the Henry coefficient was considered (0.974 and 0.905, at 37 and 33 °C respectively). The value of kLa was determined as a function of broth volume and aeration rate (kLa_(V, aer)_) in a preliminary DoE experiment using the same cultivation medium as for cell cultivation. An equation was determined based on the results of the preliminary experiment and was used to estimate kLa_(V, aer)_ on-line.

#### At-line and off-line measurements

At-line samples were taken every 24 h or more frequently in order to measure several process variables. pH measurement for in situ pH meter re-calibration was performed with a S47 SevenMulti pH meter (Mettler Toledo, Switzerland). Viable cell density (VCD) as well as viability were determined in triplicates by Countess automated cell counter (Life Technologies, CA). Samples were centrifuged at 1000*g* for 10 min, and cell-free supernatants were stored at −20 °C until further analysis. Metabolite concentrations were determined in duplicates by enzymatic assays (Cedex BioHT, Roche Diagnostics, Germany). Spent broth analysis to determine amino acid concentrations was performed by HPLC using OPA and FMOC in-needle derivatization and an Agilent ZORBAX Eclipse AAA HPLC column. Product titer was measured by affinity chromatography using a POROS Protein A column (Thermo Fisher Scientific, MA) and applying gradient elution.

#### Determination of product glycosylation

Cultivation samples were centrifuged at 1000*g* for 10 min (Rotanta 460 R, Hettich Zentrifugen, Germany), and the supernatant was purified using Protein A affinity chromatography. Enzymatic digestions were performed using trypsin, according to the protocol described before (Ozohanics et al. 2012; Turiák et al. 2011). UPLC-MS analysis of the antibody digest was performed on a Nexera UPLC (Shimadzu Corporation) coupled to a high-resolution micrOTOF-Q II mass spectrometer (Bruker Corporation). Chromatographic conditions were the following: reversed-phase column (Aeris Peptide 1.7-μm XB-C18 particles, Phenomenex Inc., USA) and gradient elution (solvent A 0.1 *v*/*v*% formic acid in water; solvent B 0.1 *v*/*v*% formic acid in 10 % water and 90 % acetonitrile mixture; flow rate 225 μL/min flow rate, column temperature 30 °C). Mass spectrometric conditions were the following: positive electrospray ionization mode (capillary voltage 4.5 kV; dry gas flow rate 12.0 L/min; dry temperature 200 °C; end plate offset 500 V) and scans acquired in the 140–2000 *m*/*z* range. The relative abundance of high-mannose glycoforms in the product amount expressed between two sampling events (i.e., two glycoform measurements) was calculated by using the mass balance in Eq. 1 in order to identify links between specific productivity and product quality.

1 $$ \varDelta \mathrm{relative}M5=\frac{M{5}_n\cdot {P}_n-M{5}_{n-1}\cdot {P}_{n-1}}{P_n-{P}_{n-1}} $$

## Results

The first goal of the study was to gain real-time information on cell physiology by monitoring the oxygen uptake of the culture on-line and to link this physiological parameter to the rate of product formation. The ultimate goal was then to develop a control strategy including specific productivity as a parameter and to investigate interactions between specific productivity and the glycosylation pattern of the recombinant product.

### On-line detection of the dynamics of physiological parameters during switches between nutrient limitation and excess

**Fig. 1:**
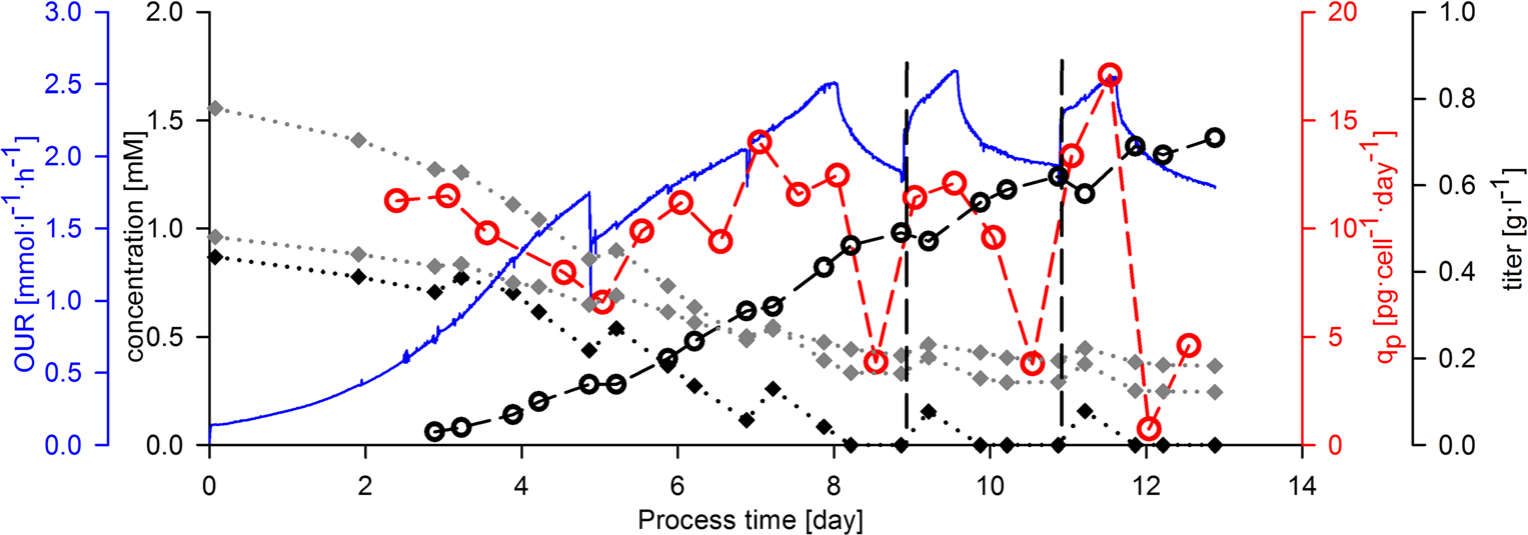
Physiological response on the switch between nutrient limitation and nutrient excess. The concentration of tyrosine (*black diamonds*) and the concentration of two aromatic amino acids, phenylalanine and tryptophan (*gray diamonds*), are represented. The on-line determined oxygen uptake rate (OUR, *blue*), the product titer (*empty black circles*), and the specific productivity (*empty red circles*) are also demonstrated. *Black dashed lines* indicate the time point of the addition of the last two bolus feeds (Color figure online)

A typical fed-batch cell culture process was performed with cell line A, using bolus feed additions every second day. OUR was monitored on-line in order to gain information on the respiratory activity of the cells (Fig. 1). On the fifth cultivation day, a temperature shift was performed leading to a decrease in the oxygen uptake of the culture. Another steep decrease in OUR was observed after the seventh cultivation day, which could not be related to any processing events. Spent broth analysis revealed that the decrease in the respiratory activity was linked to the exhaustion of tyrosine in the cultivation broth. The concentration of all other amino acids remained above the limit of detection of the measurement method during the whole cultivation (concentrations of two other aromatic amino acids which also frequently show deprivation in cell culture processes due to their low solubility in cultivation media are shown in Fig. 1). After the addition of the bolus feed (which contained tyrosine) on the ninth cultivation day, the respiratory activity of the cells recovered and OUR showed an increase for several hours. However, as tyrosine was depleted again, similar to the previously described events, OUR showed repeatedly a decline and remained on a low level until the next feeding event.

Whereas the investigation of product titer did not indicate variations in product formation during the switch between nutrient limitation and excess, the analysis of *q*_P_ revealed a steep decrease in the rate of product formation in the nutrient-limited phases (Fig. 1). However, similar to OUR, *q*_P_ recovered after the feeding events. Taken together, the exhaustion of tyrosine led to a decrease in the oxygen uptake (OUR) as well as in the productivity (*q*_P_) of the culture. The main benefit of OUR monitoring was the real-time detection of the changes in cell physiology.

### Implementation of a feeding strategy to increase *q*_P_ based on real-time physiological information

**Fig. 2:**
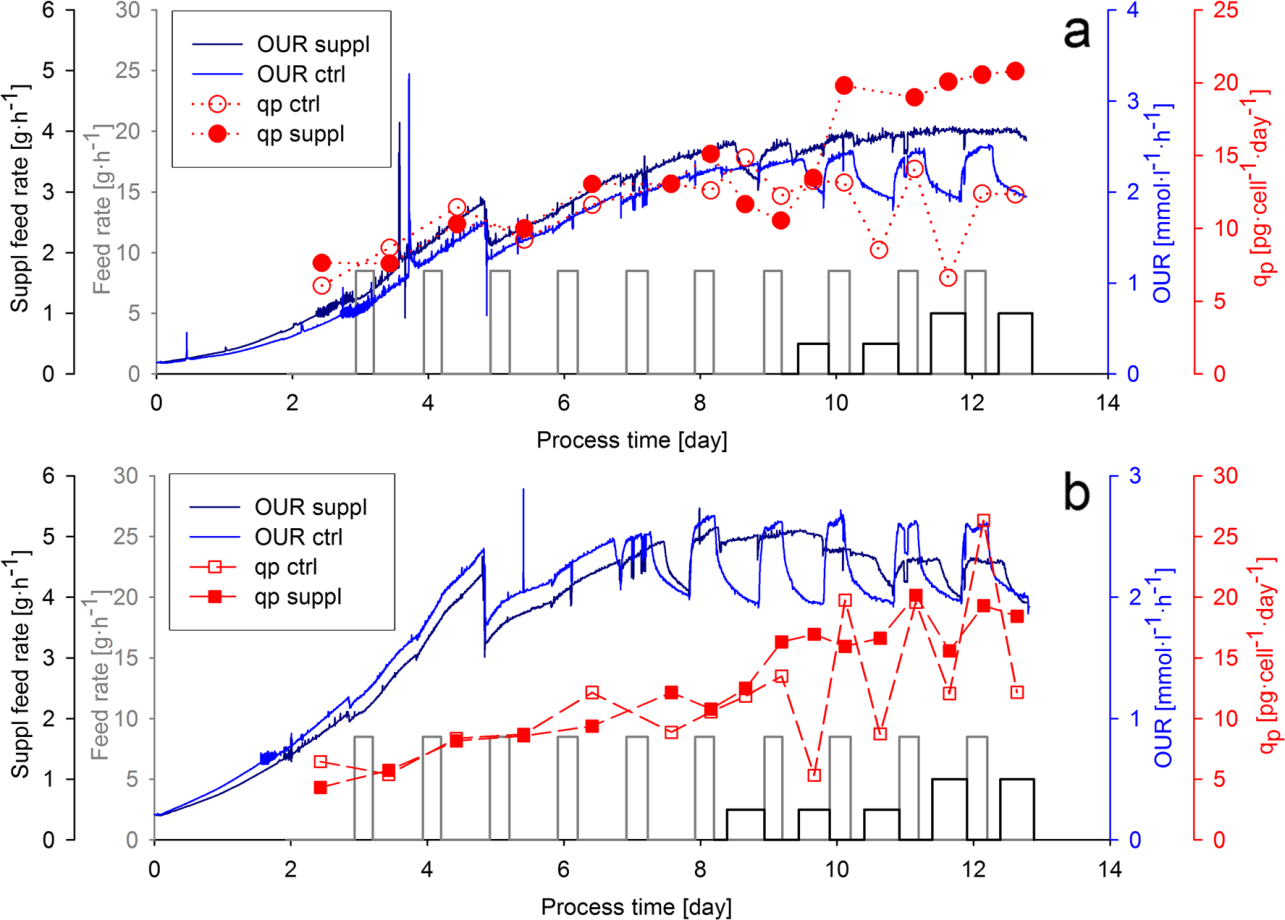
Physiological response on nutrient limitation and on the addition of a supplementary feed. The feeding rate of the daily bolus addition of the standard feed medium is shown in *gray*. *Black lines* indicate the feeding rate of the supplementary feed, started after the detection of the decrease in OUR of the control cultivation. **a** Cell line A **b** Cell line B (Color figure online)

Based on the above-discussed observations, an experiment was designed where a supplementary feed containing tyrosine (and other essential amino acids) was added to the cultivation based on the OUR signal in order to overcome nutrient limitations. The experiment was performed with two different cell lines. The supplementary feed was initiated after observing the first decrease of the OUR signal in the respective cultivation, and it was terminated at the next bolus feeding event (Fig. 2). Moreover, control cultivations were performed to obtain a similar physiological profile as shown in the previous experiment.

Interestingly, the first decrease in OUR was observed 1 day earlier in the cultivations with cell line B (day 7) compared to the cell line A cultures (day 8). Spent broth analysis (data not shown) revealed that this phenomenon was a consequence of the earlier exhaustion of tyrosine, probably due to the higher substrate uptake rates of cell line B. However, the on-line monitoring of OUR enabled to detect the earlier onset of nutrient limitations and to maintain a high specific productivity by starting the supplementary feed 1 day earlier as in the cell line A cultivation.

After the bolus feeding events, the OUR of the control cultivation (“ctrl”) with cell line A was monitored, and when the decline in OUR was detected, the supplementary feed of the supplemented cultivations (“suppl”) was started again for both cell lines. This strategy allowed to avoid nutrient limitation in the supplemented cultivation of cell line A from the first start of the supplementary feed until the end of the cultivation (data not shown). However, a decrease in OUR was observed in the supplemented cultivation of cell line B after the 11th cultivation day, suggesting the exhaustion of a further substance which was not added with the supplementary feed. The spent broth analysis revealed the exhaustion of leucine in this cultivation, which was indeed not added to the supplementary feed. The next step of process development would be to subsequently adjust the composition of the supplementary feed to the metabolic requirements of cell line B.

The time-resolved analysis of *q*_P_ revealed that product formation rate followed the pattern of the OUR signals (Fig. 2). In the control cultivations, both cell lines showed high variations in *q*_P_, in accordance with the changes in OUR. In contrast, the supplemented cultures of both cell lines A and B showed a high and nearly constant *q*_P_ after the initiation of the supplementary feeding. Thus, the real-time adjusted feeding strategy enabled us to generate different *q*_P_ patterns with two different cell lines in a single experiment. The detected differences in cell respiration as well as in *q*_P_ suggested that the cells experienced a very different physiological status in the ctrl and in the suppl cultivations. Whereas the control cultivations showed repeatedly physiological changes in nutrient limitation and excess, the addition of a supplementary feed maintained amino acid concentrations and a stable physiological status in the supplemented cultivations.

**Fig. 3:**
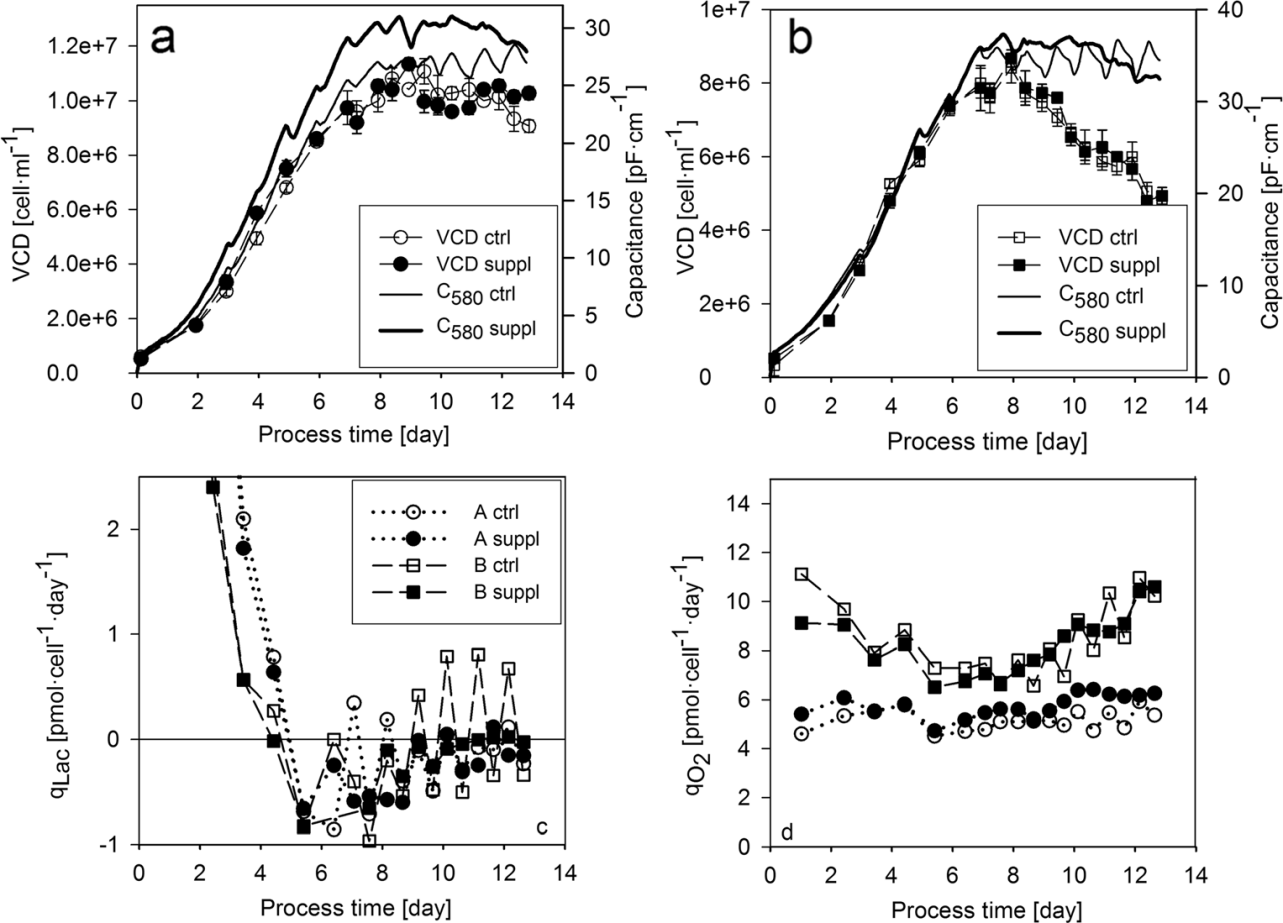
Cell growth, lactate metabolism, and respiration in the supplementary feeding experiments. **a** VCD and on-line measured capacitance for cell line A. **b** VCD and on-line measured capacitance for cell line B. **c** Specific lactate uptake rate. **d** Specific oxygen consumption rate. *Symbols* are used the same way as in Fig. 3c

Beside OUR monitoring, capacitance measurement, another on-line tool, was also performed to investigate changes in the dielectric properties of the cells during the cultivations. The capacitance signals measured at 580-kHz (C_580_) frequency showed a linear correlation to the at-line measured VCD values for both cell lines in the growth phase of the cultivations (Fig. 3a, b). The linear correlation between the capacitance signals measured at one frequency and cell density indicated a constant physiological status in this process phase. Interestingly, the capacitance signal showed a similar pattern to OUR in the control cultivations, indicating major changes in the dielectric properties of the cells as a response on the repeated switches between nutrient limitation and excess.

In order to gain insight into cell metabolism, specific lactate production rate and specific OUR (*q*_O2_) were calculated. Both cell lines switched to lactate uptake after the temperature shift performed on the fifth cultivation day (Fig. 3c). Interestingly, cell line B showed an oscillation in lactate metabolism in accordance with the phases of nutrient excess and limitation. Whereas the cells produced lactate after bolus feeding events, they switched to lactate consumption after nutrient exhaustion. Cell line A showed a different pattern indicating that the effect of nutrient limitation on lactate metabolism may be cell line dependent. The calculated *q*_O2_ values showed an oscillation in the respiratory activity of the cells (Fig. 3d), in accordance with the OUR pattern. Interestingly, the *q*_O2_ values of the two cell lines differed in a great extent, indicating differences in the metabolic activity of the two cell lines (vide supra).

**Fig. 4:**
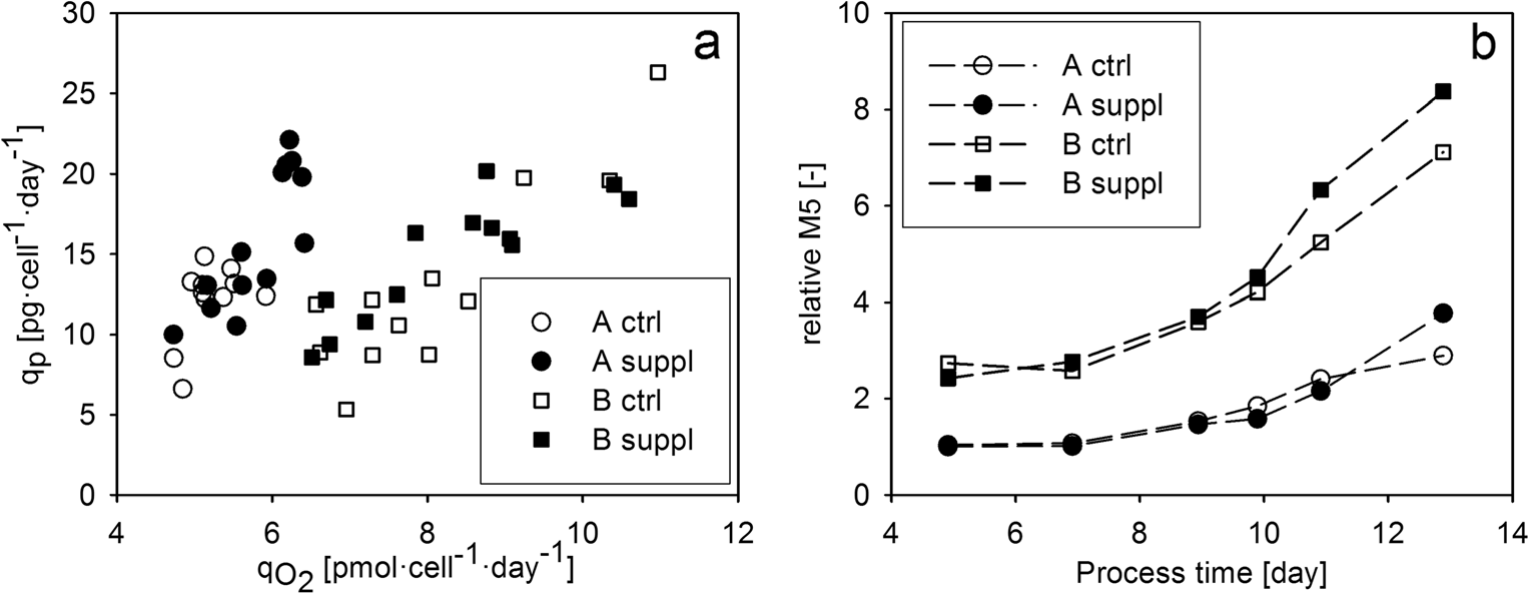
The link between respiratory activity, product formation, and product quality in the supplementary feeding experiments. **a** Specific productivity plotted against specific oxygen uptake rate. **b** Relative abundance of M5 high-mannose glycoform. The values were normalized by dividing with the value determined in first measurement point for cell line A

Specific productivity was plotted against *q*_O2_ in order to investigate the link between respiratory activity and product formation (Fig. 4a). A linear relationship was observed, verifying the tight physiological link between cell respiration and the rate of product formation in our system of interest.

An important CQA, product glycosylation—which is an N-linked biantennary oligosaccharide structure in the Fc region of the antibody—was analyzed at several time points of the cultivations. The seven most abundant glycoforms were investigated: an afucosylated high-mannose glycoform containing five mannose residues (M5) and further six glycoforms labeled according to the number of galactose in the core structure (G0, G1, and G2); each of them occurred in both fucosylated and non-fucosylated forms. The relative abundance of the glycoforms was calculated in order to investigate their distribution as a function of process time. Beside the cell line-dependent difference, a process-dependent difference was also observed in the glycosylation patterns. Generally, the relative abundance of high mannose (Fig. 4b) and other afucosylated glycoforms (G0, G1, and G2 on Fig. S1) was higher in the supplemented experiments. Both cell lines showed an increasing M5 pattern with very similar values until the tenth cultivation day in the respective control and supplemented cultivations (Fig. 4b). However, the relative abundance of M5 high-mannose glycoform was higher in both supplemented experiments, suggesting that the differences in *q*_P_ patterns led to differences in product quality. The successful adjustment of *q*_P_ enabled to identify a link between specific productivity and high mannose content. In order to investigate this link further, another experiment was conducted with cell line A, where *q*_P_ was controlled at two different levels (vide infra).

### Application of dynamic feed profiles to adjust *q*_P_ to different levels

**Fig. 5:**
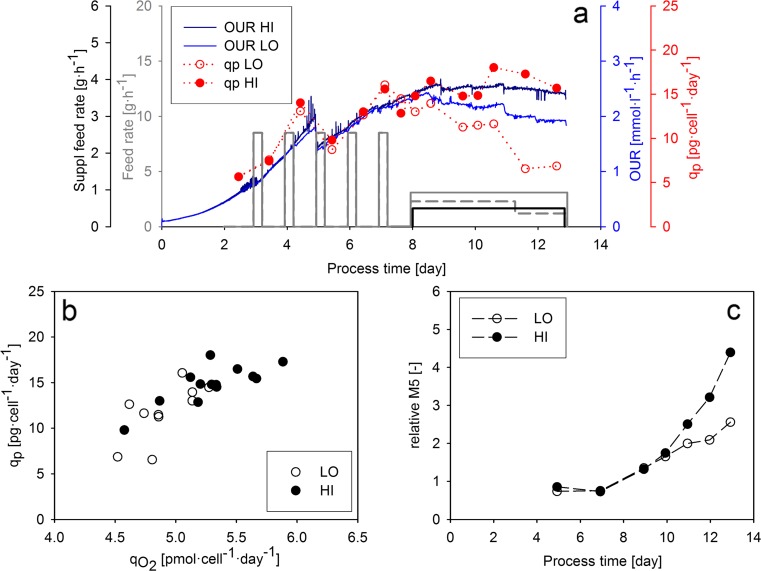
Physiological parameters, product formation, and product quality in the dynamic feeding experiments conducted with cell line A. **a** Feeding rates, oxygen uptake rate, and specific productivity (*q*_P_) in the “LO” and “HI” experiments. *Gray dashed line* indicates the feed profile of the LO experiment. *Gray* and *black continuous lines* indicate the feed profile of the HI experiment. **b** Specific productivity plotted against specific oxygen uptake rate. **c** Relative abundance of M5 high-mannose glycoform. The values were normalized by dividing the measured glycoform abundance with the value determined in the first measurement point of the bolus-fed experiment (Fig. 4b) (Color figure online)

In this experiment, feed media were added continuously to two independent cultivations performed with cell line A. The dynamic feed ramps were initiated on the eighth cultivation day based on previous observations (vide supra) to avoid any nutrient limitations. In order to achieve a high *q*_P_, both standard and supplementary feed media were added to the cultivation broth in experiment “HI.” While, in experiment “LO,” only the standard feed medium was used at a lower feeding rate to obtain a lower *q*_P_ value. Moreover, the feeding rate was reduced on the 11th cultivation day in the LO cultivation in order to trigger a decrease in *q*_P_ in the last two cultivation days (Fig. 5a). We found that both the on-line determined OUR and the calculated *q*_P_ were higher in experiment HI, indicating that the proposed strategy to control *q*_P_ by adjusting the feeding rate was successful. Moreover, both OUR and *q*_P_ immediately followed the dynamic change in the feeding rate in experiment LO on the 11th cultivation day, suggesting a strong link between feeding rate, the respiratory activity, and product formation in the nutrient-limited environment. The correlation of *q*_O2_ and *q*_P_ (Fig. 5b) in this experiment demonstrated that—similar to the bolus-fed cultivations—the rate of product formation is tightly linked to the metabolic activity of the cells in the continuous feed environment.

**Fig. 6:**
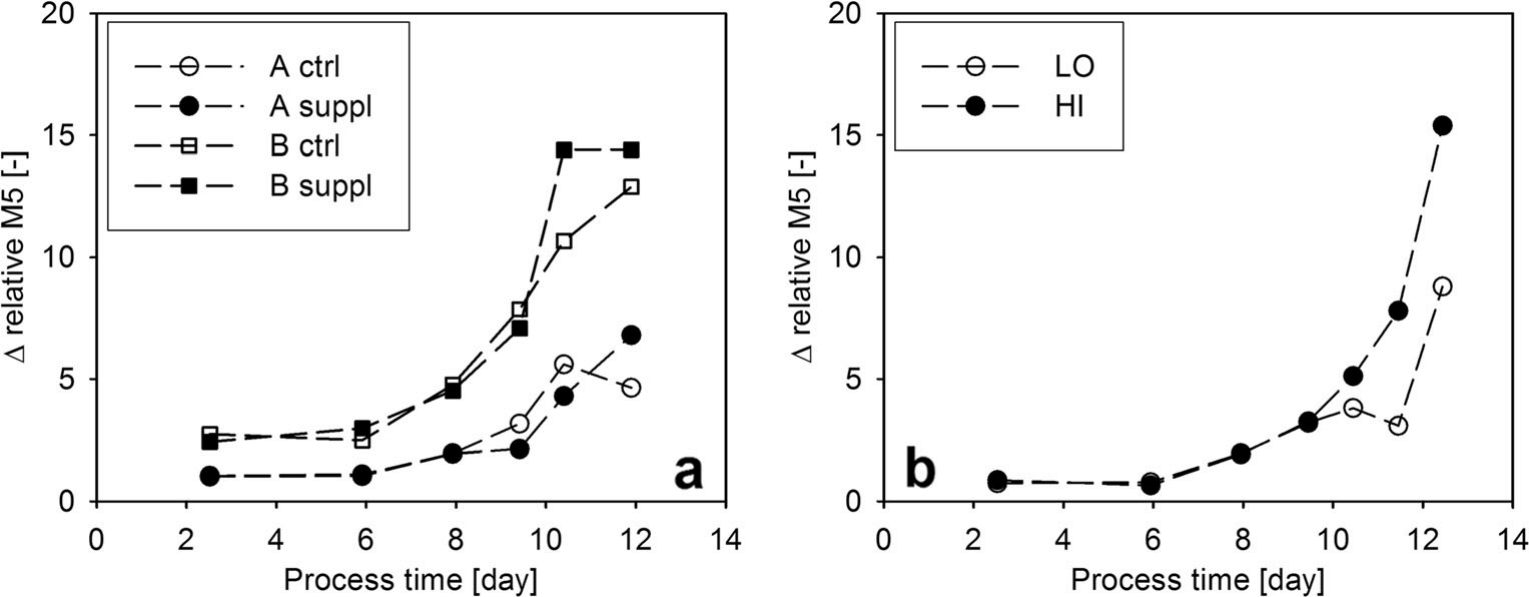
Calculated relative abundance of M5 glycoforms in the product population expressed between two sampling events. **a** Δ Relative M5 values in the bolus-fed experiments. **b** Δ Relative M5 values in the continuous feed experiments

The relative M5 value plotted as a function of process time showed a distinct response on the level of specific productivity (Fig. 5c). When the productivity of the two cultivations diverged to a great extent from each other, the relative M5 values increased in experiment HI reaching much higher values than in experiment LO. The relative abundance of G0F glycoform showed the opposite trend, and the distribution of all other glycoforms remained very similar in both cultivations (Fig. S2). These results suggest that the abundance of high-mannose glycoforms is higher when *q*_P_ is increased. As the abundance of different glycoforms was determined for the whole product population in the samples, accumulation effects may render the interpretation of glycosylation results difficult. For example, in the cultivations with low *q*_P_, a constant M5 value can be a result of the negligible effect of the low product amount on the distribution of glycoforms in the whole product population. In contrast, the high amount of new product molecules at high *q*_P_ influences the distribution of glycoforms in the respective samples to a great extent. In order to eliminate this accumulation effect, we introduced a novel calculation form by setting up a mass balance for the time ranges between two glycoform measurements (Eq. 1). Using this equation, we determined the relative abundance of high-mannose glycoforms in the product population which was expressed in the investigated time range (Δ relative M5). As shown on Fig. 6, the calculated values indicate that the abundance of M5 glycoforms is increased by high *q*_P_ values, implying the effect of the rate of product formation on product glycosylation.

### Application of *q*_P_ as a physiological factor in a multivariate experimental design

**Fig. 7:**
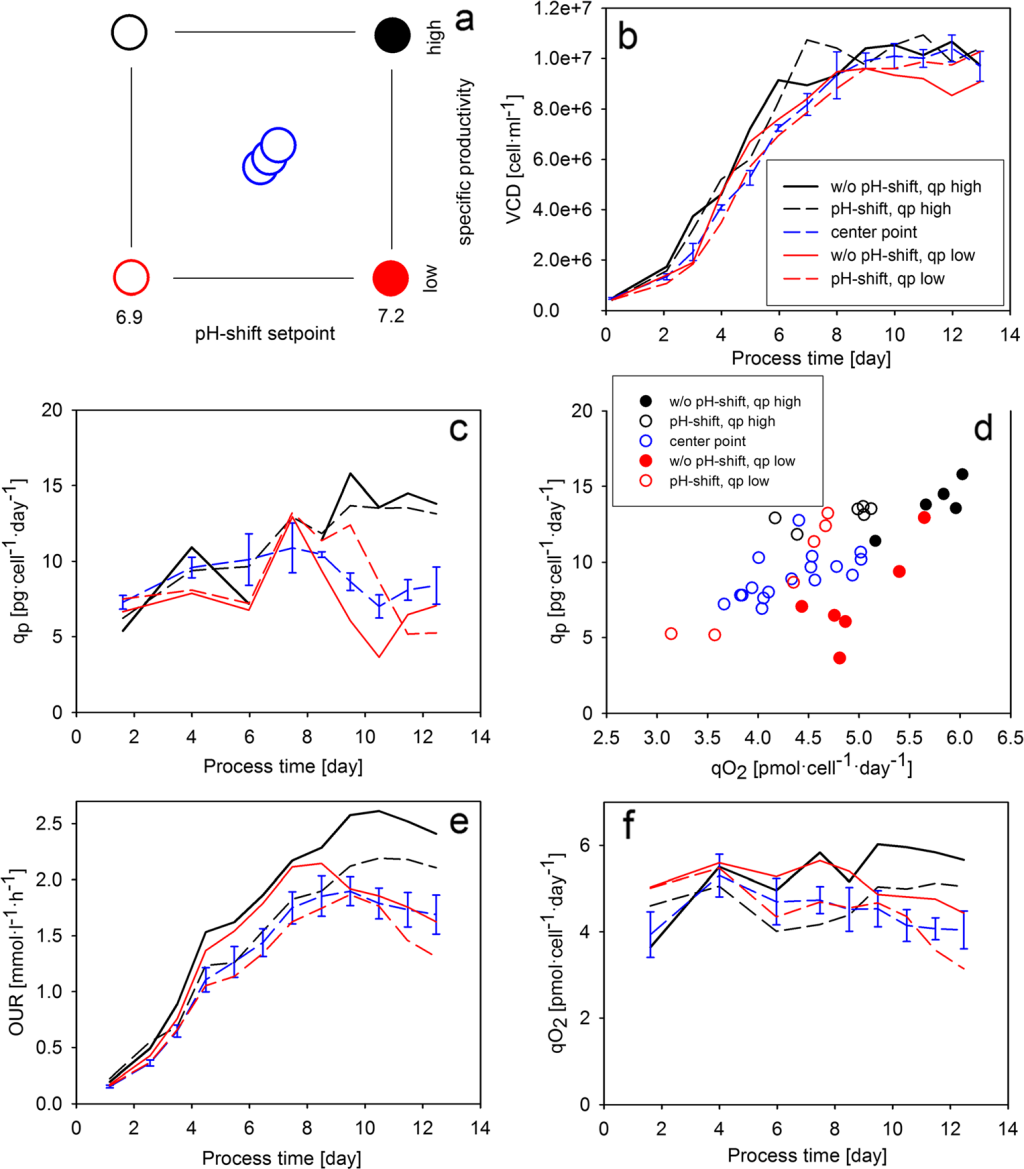
The design of experiment approach conducted with cell line A. **a** The DoE design with specific productivity as an experimental factor. **b** Specific productivity as a function of process time. **c** VCD curves. **d** Specific productivity plotted against specific oxygen uptake rate after day 7 (the values of the three center point runs are shown with *empty blue circles*). **e** Oxygen uptake rate. **f** Specific oxygen uptake rate (Color figure online)

The benefits of using physiological parameters as experimental factors in DoE designs have already been demonstrated in microbial process development (Wechselberger et al. 2012). As a proof of concept for cell culture processes, a DoE approach involving specific productivity as an experimental factor was conducted in our study (Fig. 7a). Specific productivity was controlled at three levels (“high,” “center point,” and “low”) in the last three cultivation days by applying different pre-defined continuous feeding profiles (Fig. S3a). The second experimental factor was chosen to be the pH shift setpoint, as pH was shown to affect monoclonal antibody (mAb) glycosylation (Jedrzejewski et al. 2013). The center point value of the pH shift setpoints was 7.05.

We found that the experimental factors did not affect VCD (Fig. 7b); thus, the implementation of a cell density-dependent feeding rate was not necessary. The cultivations showed a similar *q*_P_ profile until the eighth cultivation day (Fig. 7c). After the initiation of the continuous feed profiles, the specific productivity curves diverged from each other. The two “*q*_P_ high” cultivations showed a high specific productivity, as expected, until the end of the experiment. In the center point experiments, *q*_P_ started to decrease after the eighth cultivation day and then remained on a constant level in the last three days of the experiments. The highly similar *q*_P_ profile of the three center point experiments proved the reproducibility of the control strategy (Fig. S3b). Interestingly, the specific productivity in the *q*_P_ low experiments showed a pH-dependent behavior. Whereas *q*_P_ started to decrease immediately after the initiation of the continuous feeding in the “without pH shift, *q*_P_ low” cultivation, specific productivity remained high until the tenth cultivation day in the “pH shift, *q*_P_ low” run and decreased to a low level only in the last three days of the experiment. The reason behind the later decrease of product formation rate is the lower metabolic activity in pH-shifted conditions and a subsequently later onset of nutrient limitation in the “pH shift, *q*_P_ low” cultivation. Indeed, the spent broth analysis verified that tyrosine exhausted on the ninth cultivation day in the cultivation without pH shift and only on the 11th cultivation day in the “pH shift, *q*_P_ low” cultivation. However, *q*_P_ decreased to similar values in both *q*_P_ low runs, enabling the use of specific productivity as a DoE factor independent of pH in the last three days of the cultivations.

The relationship of respiratory activity and specific productivity also showed a pH-dependent pattern (Fig. 7d). Although the values of *q*_O2_ and *q*_P_ followed a linear correlation in all runs, the cultivations without pH shift formed a different cluster on the *q*_O2_–*q*_P_ plot from those where pH shift was performed. This suggested that the respiratory activity of the cells is dependent on pH. This was also confirmed by the OUR and *q*_O2_ profiles (Fig. 7e, f), where the values of the pH-shifted cultivations run lower between the time point of the pH shift (third cultivation day) and the initiation of the continuous feeds (eighth cultivation day). Nutrient availability also affected the respiratory activity of the cells. Whereas *q*_O2_ increased in the *q*_P_ high cultivations after the eighth cultivation day, the *q*_P_ low cultivations, in which the feeding rate was low, showed a decline in *q*_O2_ after the initiation of the continuous feed profile. However, the relationship between *q*_O2_ and *q*_P_ was only affected by pH shift and retained its linear nature at the different *q*_P_ levels of the DoE experiment.

**Fig. 8:**
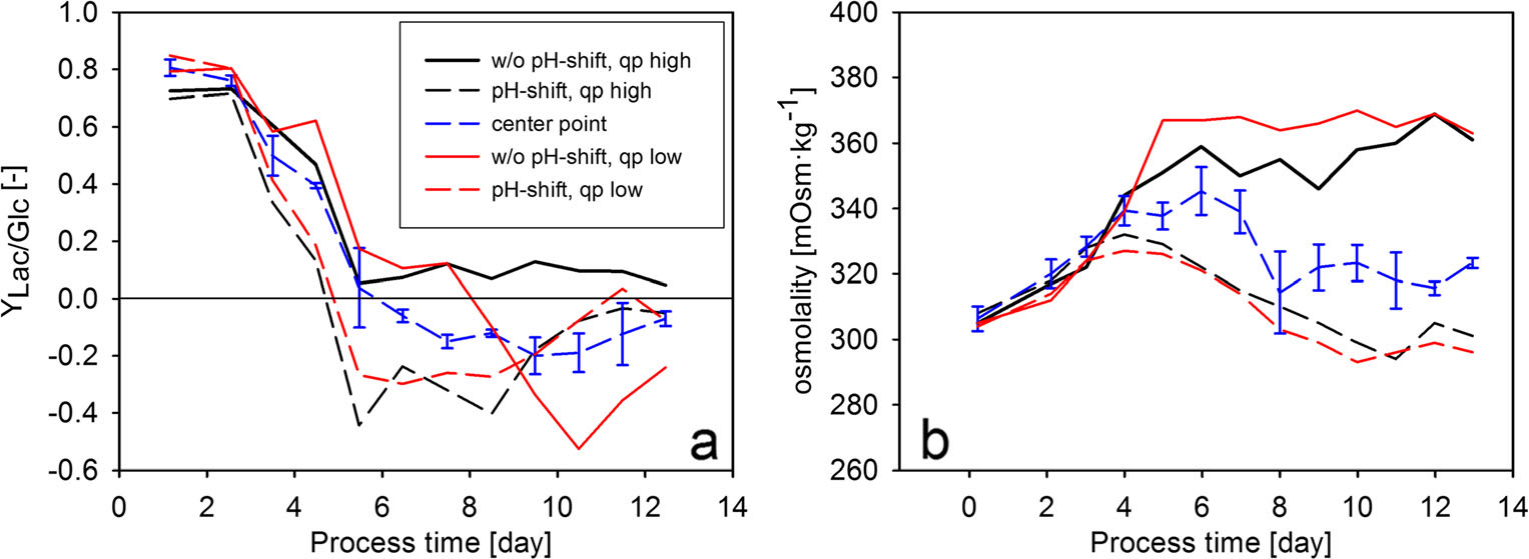
Lactate metabolism and osmolality in the DoE experiments. **a** The ratio of lactate production and glucose uptake rates. **b** Off-line determined osmolality values

In order to investigate the effect of the experimental factors on cell metabolism, the *Y*_Lac/Glc_ variable was investigated (Fig. 8a). Similar to previous observations with the same cell line (Zalai et al. 2015b), the shift in pH to 6.9 on the third cultivation day immediately affected the ratio of lactate and glucose uptake rates. Interestingly, the metabolic shift to lactate consumption was also dependent on the feeding rate in the cultivations without pH shift. Whereas the cells did not switch to lactate uptake in the cultivation with a high feeding rate, lactate consumption was observed after the eighth cultivation day in the without pH shift, *q*_P_ low. Osmolality has been shown to affect high mannose content in cell culture processes (Shi and Goudar 2014). In this study, osmolality profiles clustered according to the setpoint of the pH shift, however did not show a response on the *q*_P_ setpoint (Fig. 8b). Thus, the effect of *q*_P_ on high mannose content was not a consequence of interactions with osmolality effects.

**Fig. 9:**
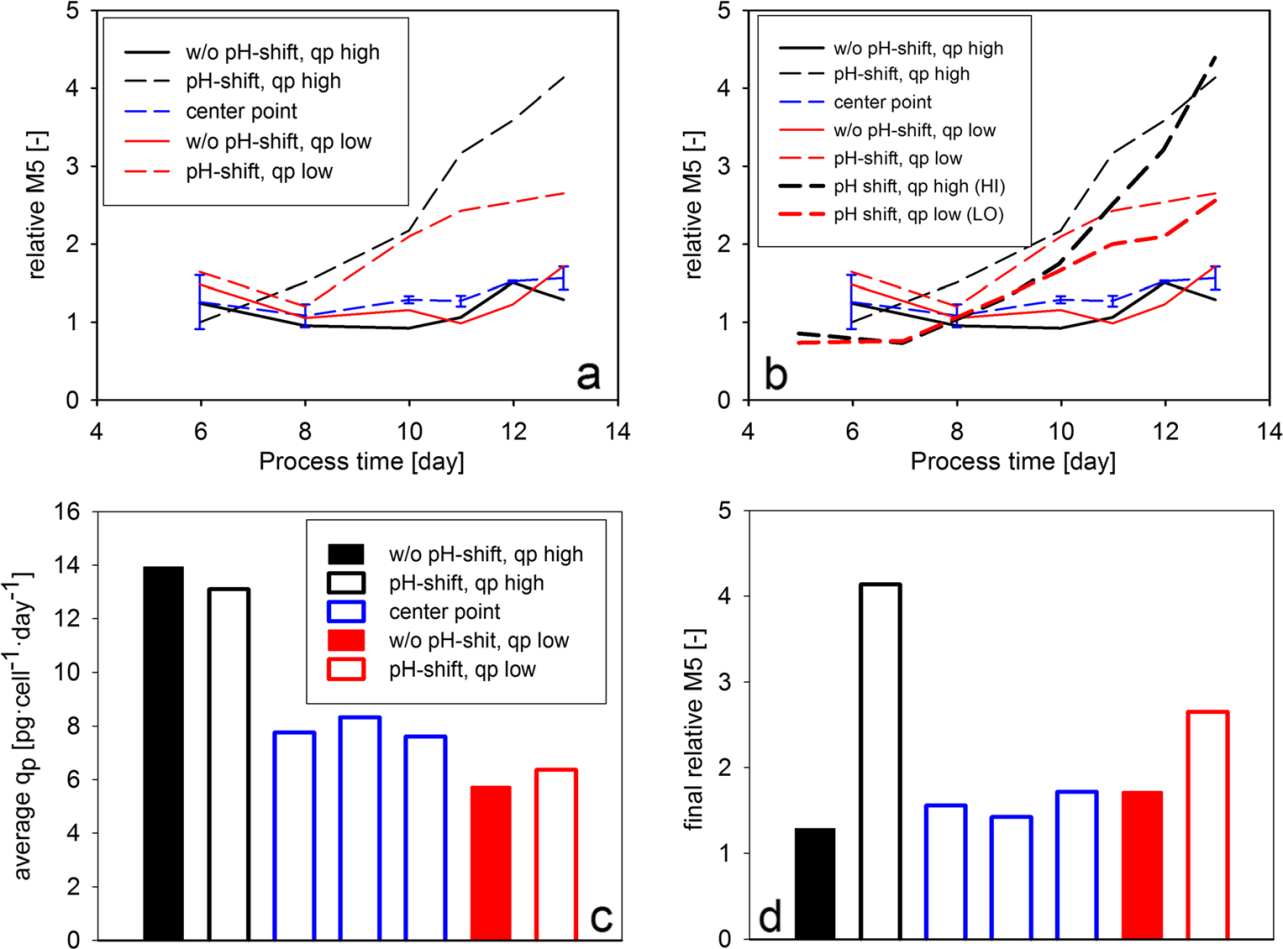
Product formation and product quality in the DoE experiments. **a** The relative abundance of M5 high-mannose glycoform as a function of process time. **b** The relative abundance of M5 high-mannose glycoform as a function of process time in the DoE as well as in the dynamic feeding experiments. **c** Average *q*_P_ values calculated from the specific productivity values in the last 3 days of the cultivations. **d** Final high mannose content. The values were normalized by dividing with the value determined in first measurement point of the bolus-fed experiment (Fig. 4b)

High mannose content was determined in the DoE cultivations in order to investigate the effect of the experimental factors on the accumulation of this pre-mature glycoform. The center point runs showed very similar high mannose profiles during the whole cultivation period (Fig. 9a). Whereas the two cultivations without pH shift showed the same profile as the center point runs, the cultivations with a pH shift to 6.9 showed elevated relative M5 values already on the tenth day of the cultivation. However, the high mannose of these cultivations diverged in the last day of the experiment, according to the *q*_P_ setpoint. The relative M5 values in the two cultivations with pH shift were comparable to the values observed in the HI and LO experiments (Fig. 9b), verifying our previous observations that the increase of *q*_P_ leads to the accumulation of pre-mature glycoforms. However, the high mannose content remained low in the “without pH shift, *q*_P_ high” cultivation, indicating that the phenomenon is pH-dependent. Taken together, the DoE experimental design enabled the identification of an interaction effect between pH and *q*_P_, which leads to elevated high mannose levels in the “pH shift, *q*_P_ high” experimental point.

In order to involve *q*_P_ as an experimental factor in the statistical evaluation of the DoE data, average specific productivity in the last three cultivation days was calculated (Fig. 9c). The values in the *q*_P_ low and the center point runs showed only a small difference, leading to an asymmetric design. Consequently, the fitted mathematical model showed a low *Q*^2^ value (0.52) as an indicator of the poor prediction capability. However, the statistical analysis identified the interaction term of pH and *q*_P_ as a significant model coefficient to explain relationship between the experimental factors and final high mannose content (Fig. 9d). This result verified the above-discussed pH dependence of the effect of *q*_P_ on high mannose content.

## Discussion

In this study, a fed-batch CHO process was characterized in order to investigate the metabolism and productivity of production cells during the cultivation. Moreover, product glycosylation was analyzed to identify the effect of process parameters and physiological parameters on product quality. Understanding the links between nutrient limitation, specific productivity, and the abundance of high-mannose glycoforms enabled the development of a control strategy to adjust this CQA in the production process.

### Changes in physiological parameters during switches between nutrient limitation and excess

The quantification of metabolic rates, specific productivity, and capacitance enabled the identification of major changes in cell physiology during the cultivation. Spent broth analysis revealed that the cause of the decrease in the metabolic activity of the cells (indicated by OUR) and in specific productivity was the depletion of tyrosine. After the addition of tyrosine with a bolus feed, both physiological parameters recovered. These results suggest that cell metabolism shows a very prompt response on the depletion and subsequently on the addition of the essential amino acid tyrosine, as indicated by the steep increase in OUR after the feeding events. Although the exact time point of the onset of tyrosine limitation could not be determined, it can be hypothesized that the steep decrease in OUR happened shortly after the limitation. Ansorge et al. reported a similar OUR pattern in a fed-batch CHO cultivation; however, the authors could not detect the depletion of amino acids with the available analytical device and thus only hypothesized that the observed decrease in OUR is a consequence of nutrient limitation (Ansorge et al. 2010).

Interestingly, the capacitance of the culture, which was also monitored on-line, showed a similar response to OUR on the switches between nutrient limitation and excess. This observation is in accordance with the results of Ansorge et al., who suggested that the observed phenomenon can be a consequence of the change in multiple physiological attributes influencing dielectric properties, such as cell size or intracellular conductivity (Ansorge et al. 2010). The changes in dielectric properties upon apoptosis induction have been recently demonstrated for the same cell line (Zalai et al. 2015c), showing that capacitance spectroscopy can be used to detect major changes in cell physiology. The exact reason for the observed variations in C_580_ in the recent study remains to be elucidated; however, it demonstrates the applicability of capacitance measurement to detect physiological changes in cell culture processes.

The change in the metabolic activity of the cells upon nutrient limitation was also verified by the *q*_Lac_ profile of cell line B in the experiments with bolus feeds (Fig. 3c). The cells produced lactate after each bolus feeding event and switched to lactate consumption when tyrosine was depleted. This observation is in accordance with literature data suggesting that the decrease of lactate production rate is a consequence of amino acid limitation (Read et al. 2013). Interestingly, cell line A did not show a similar fast response on bolus feeding events and remained in a metabolic status characterized by lactate uptake from the fifth cultivation day until the end of the cultivation (Fig. 3c). This constant metabolic status was probably a consequence of cultivation pH which was shifted to 6.9 and might have restricted metabolic fluxes to a higher extent as in cell line B. The observation that cell line A produced lactate throughout the cultivation where pH was controlled at 7.2 and a high feeding rate was maintained (Fig. 8a) supported this hypothesis further.

The kinetics of product formation was assessed by calculating *q*_P_ with a high time resolution. In accordance with several papers reporting an increase in product titer after the supplementation of limiting amino acids (Feeney et al. 2013; Read et al. 2013; Yu et al. 2011), we observed an increase in *q*_P_ after the supplementation of tyrosine (Fig. 2). However, these publications could not deliver an understanding how exactly specific productivity is affected by nutrient limitation, as *q*_P_ was calculated for either the whole cultivation period or for time windows of several days. Similarly, a *q*_P_ calculated with low time resolution (>24 h) would render the detection of dynamic changes in productivity upon nutrient depletion impossible in our study. However, by calculating *q*_P_ for sufficiently short time periods (12 h) and by monitoring other physiological variables on-line (OUR and capacitance), we were able to show a distinct response of these physiological variables on nutrient limitation as well as on feeding events.

### Control strategy to adjust specific productivity

The swift response of OUR on tyrosine depletion suggested that OUR can be used to detect nutrient limitations and to implement control strategies which respond to the limitation by the addition of the limiting substrate. Accordingly, we implemented a feeding strategy based on the real-time monitoring of OUR to supplement tyrosine. As the investigation of physiological variables with high time resolution revealed a similar response of OUR and *q*_P_ on nutrient limitation and excess, we expected that maintaining the oxygen uptake of the culture at a high level will result in high productivity. Indeed, the dynamic OUR-based feeding strategy enabled us to keep specific productivity at a constant high level throughout the cultivation (Fig. 2). OUR has been already used as an input signal for feeding strategies targeting constant cell growth or metabolism (Aehle et al. 2011b; Zhou et al. 1997). Moreover, OUR has been used as an input signal to control the addition of amino acids in mammalian perfusion processes (Aehle et al. 2012; Feng et al. 2006). However, to our knowledge, approaches to adjust *q*_P_ based on OUR have not been reported previously in the literature. The applicability of the presented control strategy was verified with two different cell lines. In the cell line B cultivation, nutrient limitation occurred one day earlier as in the cell line A cultivation, as indicated by the on-line OUR signal. However, the real-time adjusted feeding strategy enabled to respond on this difference and maintain a high *q*_P_ for cell line B, as well. This result demonstrated that a control strategy, which considers real-time physiological information, can be beneficial to respond on cell line-dependent differences during cell culture process development. Furthermore, our results support that the monitoring of OUR in cell culture process is a key Process Analytical Technology (PAT) method, which can be used to implement sophisticated control strategies (Kroll et al. 2014).

The basis of the *q*_P_ control strategy was the tight link between *q*_O2_ and *q*_P_ observed in our experimental system. The linear correlation of the two variables was observed for both cell lines, and for cell line A, the link was verified in the bolus feed as well as the continuous feed environment (Figs. 4a and 5b). These observations indicate that the metabolic activity of the cells has a vast effect on product formation. As the respiratory activity correlates to TCA flux in mammalian cells (Nargund et al. 2015; Zagari et al. 2013), a similar linear correlation of *q*_P_ and TCA cycle activity can be assumed, which has been previously observed in a fed-batch CHO cultivation (Templeton et al. 2013). Although clone-to-clone differences in *q*_O2_ have been reported to correlate with clonal variations in productivity (Ghorbaniaghdam et al. 2014), in our study, the higher *q*_O2_ of cell line B was not coupled to a higher *q*_P_. Moreover, our observations indicate that cell line-to-cell line differences in the *q*_O2_–*q*_P_ relationship have to be considered when OUR-based feeding strategies are set up.

### The effect of specific productivity on the abundance of high-mannose glycoforms

The *q*_P_ control strategy enabled to investigate the effect of product formation rate on glycosylation, one of the most CQAs of monoclonal antibodies. Glycosylation, a form of protein post-translational modification, is a result of a complex cellular process which occurs intracellularly, enclosed in the compartments of the endoplasmic reticulum and the Golgi apparatus. The formation of protein-linked glycan structures is catalyzed by enzymes of the glycosylation pathway; however, this consecutive enzymatic process is not always fully accomplished resulting in a heterogenic mixture of various glycoforms. High-mannose glycans are generated early in the glycosylation pathway, the reason why these glycoforms are considered to be pre-mature structures. Increasing *q*_P_, which was achieved by supplemented feeding in this study, led presumably to an elevated protein flux toward the glycosylation machinery. The increasing accumulation of high-mannose forms, which was shown to be associated with the elevated *q*_P_ (Fig. 5c), may be the cause of putative bottlenecks in the later phase of the glycosylation pathway. Taking the high complexity of protein glycosylation into account, the nature of these bottlenecks can be very diverse including the activation and the transport of substrates through cellular and intracellular membrane barriers or the expression level and the activity of glycosylation enzymes. For example, the identification of a bottleneck in protein translocation could help to overcome intracellular protein aggregation and enable to increase the productivity of the cells by overexpressing the enzyme responsible for protein translocation (Le Fourn et al. 2014). In a good accordance with our results, increased *q*_P_ has been reported to result in the accumulation of pre-matured oligosaccharides in mild hypothermic culture conditions (Sou et al. 2015); furthermore, high specific productivity was also discussed in the association with the increase of non-fully processed high-mannosylated glycans (Hossler 2012; Umaña and Bailey 1997). Together with the above-cited considerations, our results allow to suggest that the rate of protein production may affect the output of post-translational modification.

Interestingly, the increase of high-mannosylated glycoforms was observed when cultivation pH was shifted from 7.2 to 6.9. Combining pH and *q*_P_ in DoE experiments revealed that the phenomenon does not occur at higher pH setpoint, namely 7.05 and 7.2.

As extracellular pH influences the intracellular pH (L’Allemain et al. 1984), the activity of glycosylation enzymes might change as a result of the pH shift (Hossler et al. 2009; Rivinoja et al. 2009). Accordingly, the higher pH setpoints in the DoE experiments could lead to sufficient enzyme activities in the Golgi resolving the bottleneck of the glycosylation machinery. However, in order to elucidate the exact mechanism behind the observed phenomenon, a more comprehensive physiological characterization (e.g., proteomic and transcriptomic measurements) would be required.

The *q*_P_ control strategy presented in this study was based on the addition of the essential amino acid tyrosine at limiting levels to influence the rate of product formation. Tyrosine has been reported to be replaced by the structurally similar phenylalanine during protein translation, leading to tyrosine misincorporation and an increase in the abundance of sequence variants in tyrosine limitation (Feeney et al. 2013). These results together with ours suggest that the effect of control strategies based on nutrient limitation has to be thoroughly investigated targeting all CQAs, for example post-translational modifications as well as the primary sequence of the produced mAb.

The recent case study demonstrated the control of *q*_P_ in a fed-batch CHO process expressing a mAb. The novel control strategy was based on the tight link between substrate feeding, OUR, and specific productivity. The control of *q*_P_ at different levels enabled the investigation of links between the rate of product formation and the glycosylation pattern, mainly focusing on high-mannose glycoforms. The observed increase in the relative abundance of high-mannose glycoforms at high *q*_P_ suggests that the output of post-translational modifications is dependent on *q*_P_. Moreover, by involving specific productivity as an experimental factor in a DoE design, we could show that the link between high mannosylation and *q*_P_ is dependent on cultivation pH. Our results demonstrate that the application of PAT tools, metabolic characterization, and multivariate experimental designs can facilitate the understanding of complex interactions between process input and output parameters. Such knowledge may facilitate the development of novel control strategies to control product quality attributes in a tight pre-defined range, which is especially relevant in biosimilar process development.

## Electronic Supplementary Material

ESM 1 (PDF 610 kb)
