# Supplementary material for: A control strategy to investigate the relationship between specific productivity and high-mannose glycoforms in CHO cells
Source: Appl Microbiol Biotechnol. 2016 Feb 24;100:7011–24. doi: 10.1007/s00253-016-7380-4 (PMC4947490; doi:10.1007/s00253-016-7380-4)
Supplement: Supplementary file 1 — (PDF 610 kb) [file 253_2016_7380_MOESM1_ESM.pdf]

SUPPLEMENTARY MATERIAL

Applied Microbiology and Biotechnology

A Control Strategy to Investigate the Relationship between Specific Productivity and High Mannose Glycoforms in CHO Cells

Dénes Zalai [a,b], Helga Hevér [c], Krisztina Lovász [a], Dóra Molnár [a], Patrick Wechselberger [b,d], Alexandra Hofer [b], László Párta [a], Ákos Putics [a], and Christoph Herwig [b,d,*]

[a] Gedeon Richter Plc., Department of Biotechnology, 19-21, Gyömrői út, Budapest H-1103, Hungary

[b] Vienna University of Technology, Institute of Chemical Engineering, Research Area Biochemical Engineering, Vienna, Austria

[c] Gedeon Richter Plc., Spectroscopic Research Department, 19-21, Gyömrői út, Budapest H-1103, Hungary

[d] CD Laboratory for Mechanistic and Physiological Methods for Improved Bioprocesses, Vienna, Austria

E-mail addresses:

zalaid@richter.hu, h.hever@richter.hu, lovaasz.krisztina@gmail.com, molnardoora@gmail.com, patrick@wechs.at, l.parta@richter.hu, puticsa@richter.hu, christoph.herwig@tuwien.ac.at

*Corresponding Author: Christoph Herwig, Vienna University of Technology, Institute of Chemical Engineering, Research Area Biochemical Engineering, Gumpendorfer Strasse 1a, A-1060 Vienna, Austria. Tel: +43 1 58801 166400, Fax: +43 1 58801 166980, email: christoph.herwig@tuwien.ac.at

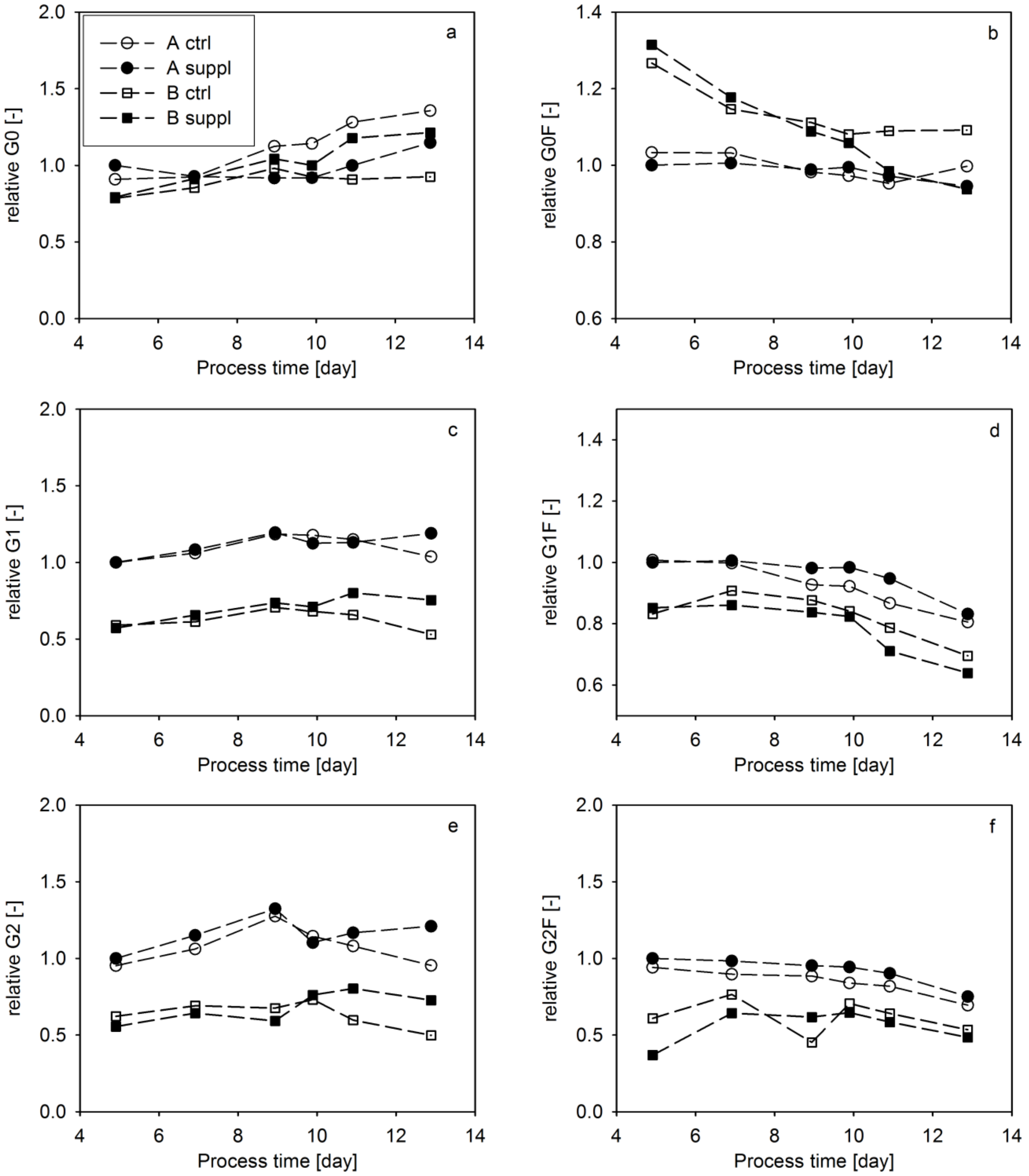

Fig. S1 The relative abundance of the major glycoforms in the bolus-fed experiments with supplementary feed (Fig. 4b). The values were normalized by dividing with the value determined in first measurement point for the respective glycoform for cell line A

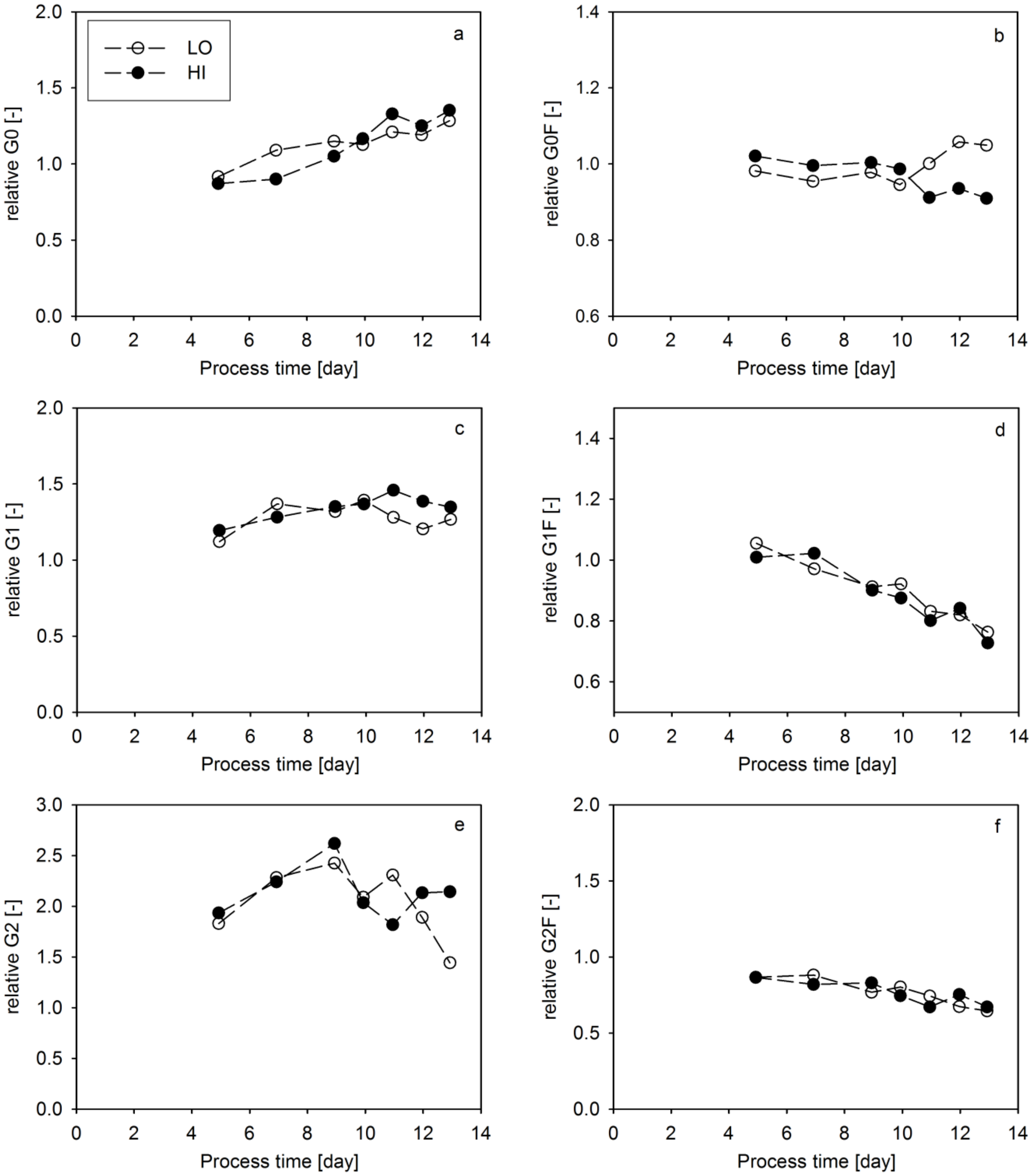

Fig. S2 The relative abundance of the major glycoforms in the dynamic feeding experiments conducted with cell line A (see Fig. 5). The values were normalized by dividing with the value determined in first measurement point of the bolus-fed experiment for the respective glycoform in the bolus-fed experiments (Fig. S1)

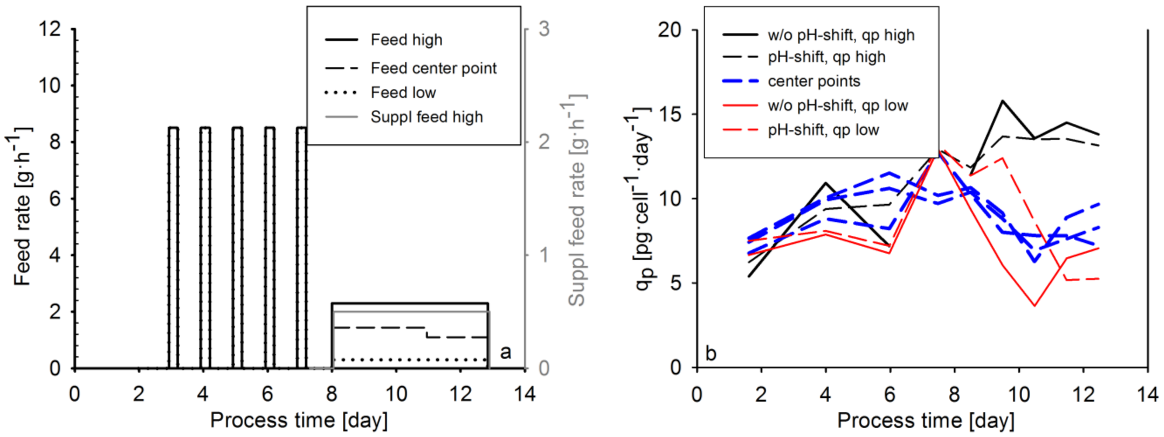

Fig. S3 a Feeding profiles in the DoE experiments. The supplementary feed was added exclusively in the "q_P high" cultivations. In the center point cultivations, the feeding rate was decreased on the 11th cultivation day based on previous observations showing an increase in q_P at a similar constant feeding rate. **b** Specific productivity profiles in the DoE experiments. Center point cultivations are represented with dashed blue lines
